# Pelvic belts and pregnancy-related pelvic girdle pain: influence on temporal and spatial gait parameters

**DOI:** 10.1080/23335432.2018.1544853

**Published:** 2018-12-05

**Authors:** Jeanne Bertuit, Clara Leyh, Véronique Feipel

**Affiliations:** a School of Health Sciences (HESAV), University of Applied Sciences and Arts Western Switzerland (HES-SO), Lausanne, Switzerland; b Laboratory of Functional Anatomy, Faculty of Motor Sciences, Université Libre de Bruxelles (ULB), Brussels, Belgium; c Laboratory of Anatomy, Biomechanics and Organogenesis, Faculty of Medicine, Université Libre de Bruxelles (ULB), Brussels, Belgium

**Keywords:** Pregnancy, gait, pelvic girdle pain, belt

## Abstract

The aims of this study were to analyze temporal and spatial parameters of gait during pregnancy in women with and without PGP, to evaluate the effect of pelvic belts on temporal and spatial gait parameters, and to compare two types of belts. A total of 46 pregnant women with PGP, 58 healthy pregnant women and 23 non-pregnant women were recruited. Temporal and spatial parameters were analysed by an walkway. Two pelvic belts for pregnant women were used. An analysis of variance for repeated measures were used. In pregnant women with PGP, compared to healthy pregnant women, gait cycle and stance phase times were lower and single support time was higher. Compared to the non-pregnant women, gait velocity and step length were lower. Stance phase and double support times were higher. During pregnancy, wearing a pelvic belt modified gait velocity, single support phase, step length, step width, stance phase and toe in/out in pregnant women with PGP. Gait adaptations in pregnant women with PGP showed nearly the same changes found in women without PGP. The belts had an effect on gait in pregnant women with PGP, probably through a biomechanical and proprioceptive mechanism.

## Background

About 50% of pregnant women suffer from pelvic girdle pain (PGP) (Vleeming et al. 2008; Robinson et al. 2010). PGP is reported as the most common cause of sick leave, with up to 32% of women having to take leave during pregnancy (Dørheim et al. 2013). The intensity of pain may be mild or quite bearable in about half of cases and very serious in about 25% of cases. Pain is localized in the posterior region of the pelvis, between the posterior iliac crest and the gluteal fold, particularly in the vicinity of the sacroiliac joint (SIJ) and it may also affect the pubic symphysis (Vleeming et al. 2008; Bertuit et al. 2017). Etiologies of PGP are multifactorial and affect the joint stability of SIJ. The ‘self-locking’ mechanism explains how shear in the SIJ is prevented by the combination of the anatomical features (form closure) and the compression generated by muscles and ligaments, which can be accommodated to the specific loading situation by a self-bracing mechanism (force closure). The tension of this specifics tissues crossing the SIJ lead to higher friction and hence stiffness (Vleeming et al. 1990). PGP seems to be related to hormonal and mechanical factors which have an impact on force closure leading to instability by a slightly larger range of movement in the pelvic joints (Aldabe et al. 2012; Kristiansson et al. 1999; Mens et al. 2009). Women with PGP suffer from considerable impairments during daily activities. Pain manifests mainly in the afternoon or evening, indicating that pain starts or increases after activities. Standing or sitting, walking and daily activities become limited in the afternoon and the evening (Bertuit et al. 2017).

A method suggested to restore pelvic stability is the use of a pelvic belt. A belt applied with even a small force should be sufficient to generate a ‘self-locking’ mechanism (Snijders et al. 1993). The compression created by the belt to the SIJ should have a stabilizing effect, by increasing the force closure, although this remains controversial (Mens et al. 2006; Soisson et al. 2015). Studies found that the use of pelvic belts decreased pain intensity by 20 mm (VAS) and made daily activities, such as walking, easier (Carr 2003; Kalus et al. 2008; Bertuit et al. 2017). One possible explanation could be linked to a decreased muscle activity (Park et al. 2010; Jung et al. 2013) and a release of tension in the ligaments (the sacrospinous, sacrotuberous and the interosseous sacroiliac ligaments) during the use of a belt (Sichting et al. 2014). In a previous study, a pelvic belt was shown to be efficient for altering muscle activation patterns (Oh 2014). It is also likely that the belt has an effect on motor activities such as gait. Wearing a belt would provide postural support which would significantly improve gait stability (Krkeljas 2017).

Gait undergoes changes during pregnancy in order to obtain a safe gait and to reduce the risk of falling (Bertuit et al. 2015). To date, only one study has evaluated biomechanical parameters during gait in pregnant women with PGP (Wu et al. 2008). The results illustrated that women with PGP displayed a modification of velocity according to gait speed and kinematics alterations at the pelvis, spine and thorax. However, this study had a small sample and did not evaluate temporal and spatial gait parameters. We could hypothesize that pain biomechanically changes the gait. However, this does not seem to be the case for all parameters. Since gait is an important daily activity, which is difficult to achieve for pregnant women with PGP, and considering the limited amount of literature on the subject, it is essential to improve and enrich our knowledge about any possible motor changes—such as gait—for pregnant women with PGP. If the gait of pregnant women with pelvic pain is altered, it would be interesting for clinical practice to be able to assess whether we can influence the biomechanical parameters of this motor activity with the use of a pelvic belt. Gait could be facilitated, making the belt a useful and valid tool for treatment and prevention. Belts are easy to use and without side effects, and could be well-suited for pregnant women with PGP . However, many types of belts have not yet been assessed, making it difficult to use them as part of an evidence-based practice.

The first objective was to analyze by a cross-sectional study the temporal and spatial parameters of gait in pregnant women with and without PGP. The second objective was to evaluate the effect of pelvic belts on those temporal and spatial gait parameters during pregnancy. The last objective was to compare two types of belts (narrow and flexible and broad and rigid).

## Methods

### Participants

The characteristics of the three groups are presented in Table 1. For the first group (PGP-PW), 66x pregnant women with PGP aged 25 to 35 years were recruited. The inclusion criteria were: women from the 18^th^ week of pregnancy, with pain in the sacroiliac joints and/or pubic region—as verified by a set of tests during clinical examination (posterior pelvic pain provocation test, Patrick Faber’s test, Trendelenburg modified test and active straight leg raise test) (Mens et al. 1996; Albert et al. 2000). The exclusion criteria were: the presence of lumbopelvic pain before pregnancy, as well as other pathologies involving gait problems, surgery of the lumbar spine, pelvis, hips or knees, fractures, pain radiating below the knee, tumours or active inflammation in the lumbopelvic region, presence of known anomalies of the spine, and rheumatic diseases. Twin pregnancies and pregnancies with complications were also exclusion criteria. Participants were randomized by throwing the dice into groups (A1/A2/B). Group A included 38 women who wore a belt during pregnancy but not during gait evaluation. Belts were used during 9 (± 5) weeks of pregnancy. Seventeen women formed A1 using belt 1 (22 women with 5 drop-outs) and 21 women formed A2 using belt 2 (24 women with 3 drop-outs). Group B included 20 women who did not wear a belt. There were 12 drop-outs, which reduced the number of women in this group to 8. Thus 46 women completed the study.

**Table 1.** Characteristics of the study samples

						*Week of pregnancy*				*Pain – VAS (mm)*			
*Groups*			*Number*	*Age (years)*	*Height (cm)*	*T1*	*T2*	*T1-T2*	*Mass gain (kg)*	*T1*	*T2*	ES	95% CI
PGP-PW	A	*A1*	17	29 (5)	161 (4)	28 (4)	36 (1)	8 (4)	13 (5)	60 (20)	30 (40)	0.9	[0.2, 1.7]
		*A2*	21	30 (5)	162 (5)	26 (5)	35 (1)	9 (5)	12 (4)	60 (20)	50 (30)	0.4	[−0.2, 1.0]
		*A1+ A2*	38	30 (5)	162 (5)	27 (5)	36 (2)	9 (5)	12 (5)	60 (20)	40 (30)	0.8	[0.3, 1.3]
	B		8	29 (5)	163 (6)	27 (6)	36 (2)	10 (7)	12 (2)	50 (30)	50 (30)		
	A + B		46	30 (5)	162 (5)	27 (5)	36 (2)	9 (5)	12 (4)	/	/		
H-PW			58	29 (5)	166 (6)	33 (4)	/		10 (4)	/	/		
CG			23	27 (5)	168 (6)	/	/		/	/	/		

PGP-PW: pregnant women with PGP, H-PW: healthy pregnant women, CG: control group A: women with belt during pregnancy (A1: women with belt 1, A2: women with belt 2) – B: women without belt during pregnancy T1: first evaluation, T2: second evaluation ES: Effect Size

For the second group (H-PW), 58 healthy pregnant women, aged between 24 and 31 years were included, from the 18^th^ week of pregnancy. The exclusion criteria were similar to PGP-PW, with the addition of the presence of lumbopelvic pain during pregnancy, and pain in the sacroiliac joints and/or pubic area.

The third group, corresponding to the control group (CG), included 23 non-pregnant women of the same age range, free from pelvic pain, and without any previous surgery.

All subjects gave written informed consent prior to participation in the study, which was approved by the Ethics Committee of University and Hospital Erasme (Be) (number P2011/017).

### Equipment used

The spatial and temporal parameters of gait were measured using an walkway (GAITRite Gold, CIR Systems, PA, USA, length: 6.1 m, width: 61 cm). Embedded pressure sensors form a horizontal grid. As the subject walks over the walkway, sensor activation enables the collection of spatial and temporal gait parameters. Data is sampled at a frequency of 100 Hz. Spatial and temporal gait characteristics were processed and stored using GAITRite GOLD, version 3.9 software.

**Figure 1.**
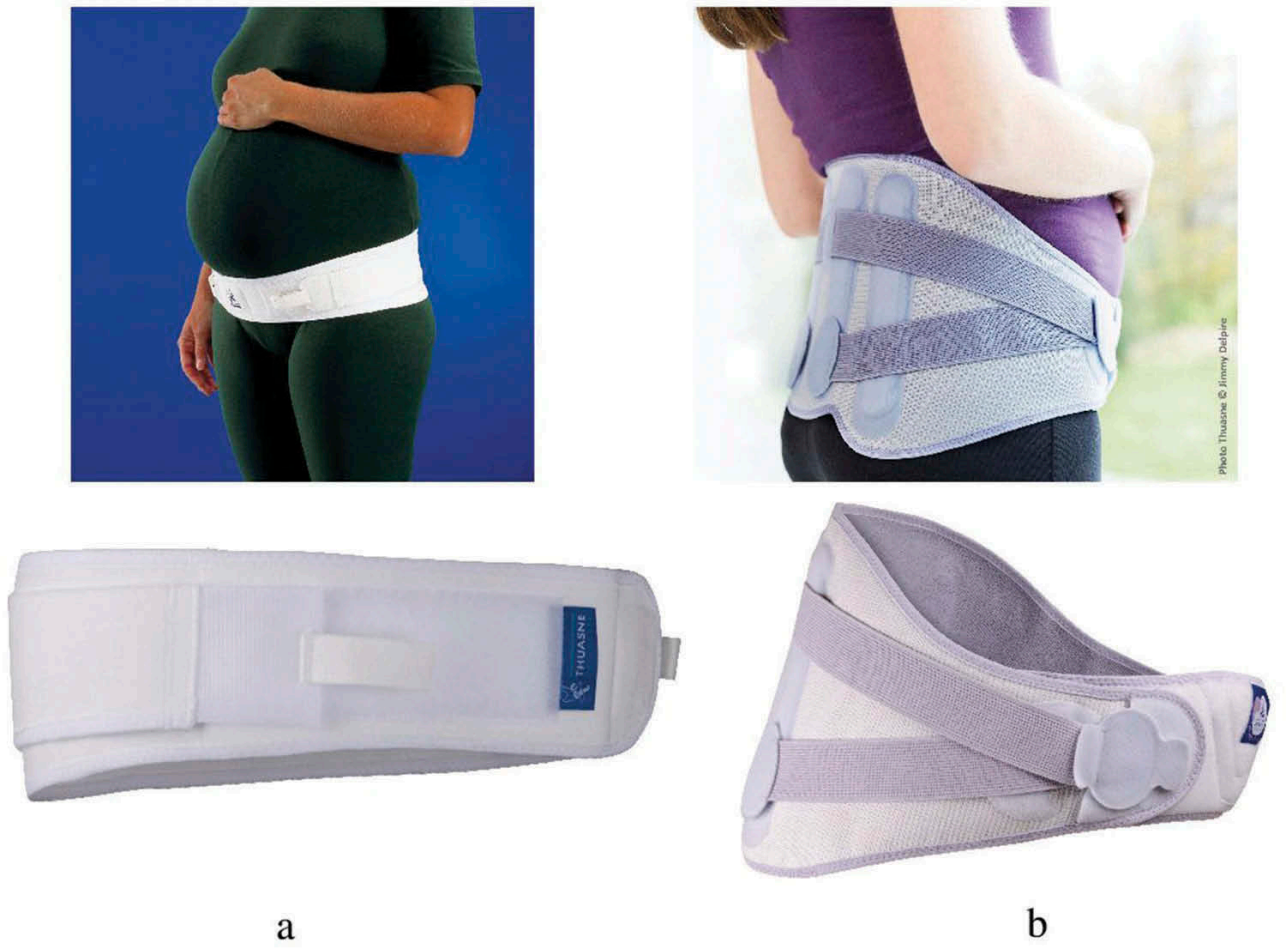
Pelvic belt. www.thuasne.com

Two pelvic belts for pregnant women were used:

- Belt 1 (Ortel-P, Thuasne) (Figure 1(a)). This belt is narrow and flexible. The belt can be placed in two positions: high position (at the level of the anterior superior iliac spine) or low position (at level of the pubic joint). Women first had the belt adjusted to their body, and then modified the belt pressure with the help of elastic Velcro systems on each side.
- Belt 2 (LombaMum, Thuasne) (Figure 1(b)). This belt is broad and rigid with metal reinforcements in the lumbar area. It allows only one position but a sophisticated Velcro system makes it possible to adjust tension to a number of different levels.

Instructions on the use of belts were provided to patients during an information session. Women were instructed to freely choose the tension (Pel et al. 2008), and belt position (Vleeming et al. 1992; Snijder et al. 1993). In order to control the use of the belt daily, women had to keep a diary and record the number of hours per day or the belt was worn.

### Data collection

Because it is known that pregnant women use specific strategies to adapt to changes in gait speed (Wu et al. 2008), the motor task consisted of nine gait trials, three at each speed. Each participant was invited to walk barefoot on the walkway. Gait speeds were self-selected, but standardized instructions were used. A rest period was allowed between trials. First, subjects were invited to walk at their preferred speed. Then, subjects walked at fast and slow speeds. Their order was randomized. The instructions for fast speed were: ‘Walk as fast as possible, as if you wanted to catch a bus’ and the instructions for slow speed were: ‘Walk slowly, as if you were shopping’. To counter the methodological bias of acceleration and deceleration in gait, participants started walking 2 m ahead of the walkway and finished the trial 2 m after the end of the walkway.

Each group performed a gait assessment without a belt (T1). The women in the PGP-PW group wore a belt for 9 (+- 5) weeks. Both groups were evaluated a second time without belt between the 34th and 38th week (T2).

### Data processing

During data collection, information were collected: age, height, weeks of pregnancy (T1/T2) and weight (T1/T2) to determine the mass gain between the two evaluations. Intensity of pain threshold was evaluated with a visual analog scale (VAS) (Table 1).

The following dependent variables were analysed: step length (m), step width (m), and toe in/out angle (degrees) for spatial parameters and gait velocity (m/s), gait cycle time (s), stance time (% of gait cycle), single and double support times (% of gait cycle) for temporal parameters. The intra-individual variability of these parameters was also evaluated by means of their individual standard deviations, computed for each speed across all cycles sampled at that speed.

### Statistical analysis

All statistical procedures were conducted using Statistica 5.0 software for Windows. To investigate the normal distribution of the data we used the Kolmogorov-Smirnov test. All scores were found to be normally distributed. A Student’s t-test for paired samples was not significantly different between sides: data of left and right foot were thus averaged. An analysis of variance for repeated measures (ANOVA) was performed for the comparison of the dependent variables between different speeds and time points (within group factor) and groups (between groups factor). When a significant effect was found, the LSD *post hoc* test was applied. The statistical level of significance was set at 0.05.

Effect sizes (Cohen’s d with 95% confidence intervals, CI) of differences between groups and test moments were computed and interpreted according to Cohen (1977) and Coe (2002).

## Results

- Characteristics of the subjects:

No statistical differences were observed between groups for age, height, weeks of pregnancy, mass gain, and level of pain. No relevant linear correlation was found between the two latter variables and spatio-temporal parameters (−0.47 < r < 0.50). For this reason, ANCOVA was not applied.

- PGP-PW gait evaluation (Table 2):

**Table 2.** Mean (SD) temporal and spatial parameters of according to speed and groups

	Speed	CG	H-PW	PGP-PW (A + B)	*ANOVA P-values Post-hoc*			
					*Speed*	*Groups*	*PGP-PW Vs H-PW*	*PGP-PW Vs CG*
Gait velocity	S	0.81 (0.13)	0.71 (0.21)	0.73 (0.18)	*< 0.001*	*< 0.001*	*0.652*	*< 0.001*
(m/sec)	P	1.26 (0.13)	0.99 (0.16)	1.02 (0.21)				
	F	1.76 (0.22)	1.4 (0.24)	1.40 (0.47)				
Gait cycle time	S	1.39 (0.18)	1.49 (0.32)	1.41 (0.24)	*< 0.001*	*0.005*	*0.040*	*0.145*
(sec)	P	1.07 (0.07)	1.20 (0.15)	1.16 (0.11)				
	F	0.89 (0.07)	0.98 (0.11)	0.93 (0.11)				
Stance	S	61 (1)	63 (3)	62 (3)	*< 0.001*	*< 0.001*	*< 0.001*	*0.001*
(%)	P	59 (1)	61 (2)	60 (2)				
	F	57 (2)	59 (2)	58 (2)				
Single support	S	39 (1)	37 (5)	37 (2)	*< 0.001*	*0.006*	*0.004*	*0.947*
(%)	P	41 (1)	39 (2)	40 (2)				
	F	43 (1)	41 (2)	43 (3)				
Double support	S	22 (2)	28 (7)	26 (4)	*< 0.001*	*< 0.001*	*0.087*	*< 0.001*
(%)	P	17 (2)	24 (8)	21 (3)				
	F	14 (3)	19 (4)	16 (3)				
Step length	S	0.55 (0.04)	0.51 (0.07)	0.50 (0.07)	*< 0.001*	*< 0.001*	*0.715*	*< 0.001*
(m)	P	0.67 (0.05)	0.58 (0.06)	0.58 (0.08)				
	F	0.78 (0.06)	0.67 (0.05)	0.67 (0.09)				
Step width	S	0.08 (0.03)	0.10 (0.03)	0.10 (0.03)	*0.713*	*0.087*		
(m)	P	0.08 (0.02)	0.10 (0.03)	0.10 (0.03)				
	F	0.09 (0.03)	0.10 (0.03)	0.10 (0.02)				
Toe in/out	S	4 (5)	5 (6)	7 (5)	*< 0.001*	*0.159*		
(degrees)	P	3 (5)	4 (4)	5 (5)				
	F	2 (4)	3 (4)	4 (5)				

PGP-PW: pregnant women with PGP, H-PW: healthy pregnant women, CG: control group S: slow, P: preferred, F: fast

PGP-PW, when compared to H-PW, displayed a 5% (*p* = 0.040) significantly shorter gait cycle and a 2% (*p* < 0.001) shorter stance phase. In addition, the single support phase was longer, by 3% (*p* = 0.004).

When compared to the CG, PGP-PW walked more slowly (−10% at slow speed, −19% at preferred speed and −20% at fast speed—*p* < 0.001—interaction groups x speeds: *p* = 0.005). The stance phase was longer by 2% (*p* = 0.001) and the double support phase by 18% (*p* < 0.001). PGP-PW walked with smaller steps when compared to the CG, representing a 9% % smaller step length at slow speed, 13% at preferred speed and 14% at fast speed (*p* < 0.001 – interaction groups x speeds: *p* < 0.001).

- Effect of a pelvic belt during pregnancy for PGP-PW (Table 3):

**Table 3.** Effect of belt – Mean (SD) temporal and spatial parameters of according to speed, times and groups

		Women with belt (A)			Women without belt (B)			*p value*	Belt 1 (A1)			Belt 2 (A2)			*p value*
	Speed	T1	T2	*p value*	T1	T2	*p value*	*A/B*	T1	T2	*p value*	T1	T2	*p value*	*A1/A2*
Gait velocity	S	0.73 (0.17)	0.74 (0.15)	*0.011*	0.73 (0.21)	0.80 (0.21)	*0.928*	*0.920*	0.78 (0.20)	0.78 (0.18)	*0.196*	0.69 (0.13)	0.71 (0.11)	*0.022*	*0.866*
(m/sec)	P	1.02 (0.23)	0.93 (0.17)		1.00 (0.14)	0.97 (0.17)			1.05 (0.20)	0.93 (0.16)		1.00 (0.25)	0.93 (0.19)		
	F	1.42 (0.47)	1.27 (0.27)		1.32 (0.48)	1.26 (0.27)			1.30 (0.58)	1.24 (0.25)		1.52 (0.34)	1.30 (0.28)		
Gait cycle time	S	1.41 (0.24)	1.36 (0.14)	*0.271*	1.41 (0.23)	1.35 (0.26)	*0.917*	*0.944*	1.36 (0.20)	1.34 (0.15)	*0.145*	1.46 (0.27)	1.36 (0.13)	*0.856*	*0.775*
(sec)	P	1.16 (0.12)	1.20 (0.09)		1.15 (0.09)	1.17 (0.09)			1.15 (0.12)	1.20 (0.08)		1.16 (0.12)	1.19 (0.10)		
	F	0.92 (0.11)	1.00 (0.11)		0.97 (0.13)	1.02 (0.12)			0.94 (0.09)	1.02 (0.09)		0.90 (0.12)	0.99 (0.13)		
Stance	S	62 (3)	63 (2)	*0.001*	62 (2)	62 (2)	*0.060*	*0.467*	62 (2)	62 (2)	*0.051*	63 (3)	63 (1)	*0.003*	*0.484*
(%)	P	60 (2)	61 (2)		60 (0)	61 (1)			60 (2)	61 (2)		60 (2)	61 (2)		
	F	58 (2)	59 (2)		57 (3)	59 (2)			58 (1)	59 (2)		58 (2)	59 (2)		
Single support	S	37 (2)	37 (2)	*0.028*	38 (2)	38 (2)	*0.448*	*0.957*	38 (2)	38 (2)	*0.532*	37 (2)	37 (1)	*0.028*	*0.604*
(%)	P	40 (2)	39 (2)		40 (0)	39 (1)			40 (2)	39 (2)		40 (2)	39 (2)		
	F	42 (3)	41 (2)		43 (2)	41 (2)			43 (4)	41 (2)		42 (2)	41 (2)		
Double support	S	26 (4)	26 (3)	*0.741*	25 (4)	24 (5)	*0.432*	*0.889*	25 (4)	25 (4)	*0.305*	27 (4)	26 (3)	*0.652*	*0.082*
(%)	P	21 (3)	23 (4)		20 (1)	21 (3)			20 (3)	22 (4)		21 (3)	23 (4)		
	F	16 (4)	18 (3)		16 (3)	19 (3)			16 (2)	18 (3)		17 (4)	18 (3)		
Step length	S	0.50 (0.07)	0.50 (0.06)	*< 0,001*	0.49 (0.06)	0.51 (0.06)	*0.261*	*1,000*	0.51 (0.08)	0.52 (0.06)	*0.034*	0.50 (0.06)	0.48 (0.06)	*0.006*	*0.525*
(m)	P	0.58 (0.08)	0.55 (0.07)		0.57 (0.04)	0.56 (0.06)			0.59 (0.07)	0.56 (0.07)		0.57 (0.09)	0.54 (0.08)		
	F	0.67 (0.09)	0.63 (0.08)		0.67 (0.08)	0.62 (0.08)			0.67 (0.10)	0.63 (0.09		0.67 (0.09)	0.63 (0.08)		
Step width	S	0.10 (0.04)	0.12 (0.03)	*0.002*	0.10 (0.03)	0.10 (0.04)	*0.370*	*0.301*	0.09 (0.04)	0.11 (0.03)	*0.027*	0.11 (0.03)	0.12 (0.03)	*0.011*	*0.310*
(m)	P	0.10 (0.03)	0.11 (0.04)		0.08 (0.04)	0.10 (0.03)			0.09 (0.03)	0.10 (0.03)		0.11 (0.03)	0.12 (0.04)		
	F	0.10 (0.02)	0.11 (0.03)		0.10 (0.03)	0.10 (0.04)			0.10 (0.02)	0.11 (0.04)		0.10 (0.03)	0.12 (0.03)		
Toe in/out	S	7 (5)	8 (5)	*0.002*	5 (6)	5 (5)	*0.672*	*0.240*	6 (5)	7 (4)	*0.023*	8 (5)	9 (5)	*0.023*	*0.113*
(degrees)	P	6 (5)	7 (5)		4 (4)	4 (6)			4 (4)	6 (5)		7 (5)	8 (5)		
	F	4 (5)	5 (4)		3 (5)	4 (5)			3 (4)	4 (4)		5 (5)	5 (4)		

S: slow, P: preferred, F: fast T1: First evaluation, T2: second evaluation

From a transversal perspective, no differences appeared between groups A (who wore a belt during pregnancy) and B (who did not). However, several parameters changed between T1 and T2 for the group A (longitudinal perspective): gait velocity decreased by 7% (*p* = 0.011), single support phase by 2% (*p* = 0.028) and step length by 4% (*p* < 0.001). Stance phase increased by 2% (*p* = 0.001), step width by 1 ± 0.3 cm, or 13% (*p* = 0.002) and the toe in/out by 1 ± 5 degrees (*p* = 0.002). The effect sizes were however small (d ≤ 0.5), except for the increase in step width, for which the effect size was medium (d = 0.57, 95%CI [0.11; 1.02]).

For group B, who did not wear a belt during pregnancy, none of the parameters changed.

- Effect of the type of belt (Table 3):

No differences appeared between the group with belt 1 (A1) and the group with belt 2 (A2). However, during pregnancy (T1/T2) several differences appeared in each group: for pregnant women with belt 1 (A1), three parameters were modified. Step length decreased by 4% (*p* = 0.034), step width increased by 1 to 2 cm (*p* = 0.027 – Interaction groups x speeds: *p* = 0.03), with a medium effect size at fast speed (d = 0.57, 95%CI [0.11 1.02]). Toe in/out angle increased by 2 ± 4 degrees (*p* = 0.023).

For pregnant women with belt 2 (A2): gait velocity decreased by 7% at preferred speed and by 14% at fast speed (*p* = 0.022 – Interaction groups x speeds: *p* = 0.02), with a medium effect size at fast speed (d = 0.71, 95%CI [−0.06 0.85]). Step length decreased by 5% (*p* = 0.006). Single support time decreased by 2% (*p* = 0.028) and stance phase increased by 1% (*p* = 0.003). For the two latter changes, a medium effect size was found at preferred and fast speeds (d = 0.5, maximal 95%CI [0,04 0,96]). Step width increased by 1 to 2 cm according to speed (*p* = 0.011 – Interaction groups x speeds: *p* = 0.03) (d = 0,67, 95%CI [0,06 0,85] at fast speed) and toe in/out angle by 1 ± 5 degrees (*p* = 0.023).

For the remaining comparisons, effect sizes were however small (d ≤ 0.5).

- Intra-individual variability of gait parameters:

The individual standard deviations of gait parameters were low, averaging 0.02 s for temporal parameters and 0.02 m for spatial parameters. No differences were found between groups, except for gait cycle time variability, which was significantly higher (*p* = 0.035) in both groups of pregnant women (0.02 ± 0.01 sec) when compared to controls (0.01 ± 0.00 sec).

## Discussion

Bertuit et al. (2015) observed that the gait speed of healthy pregnant women (0.99 ± 1.06 m/s) was lower by 22% compared to non-pregnant women (1.26 ± 1.13 m/s). Although the differences in walking speed between pregnant and null-gravidae are equivocal, our studies show pregnant women with PGP (1.02 ± 0.21 m/s) walked at a 19% slower pace than non-pregnant women (1.26 ± 0.13m/s) similarly to those of Bertuit et al. (2015). Both pregnant women with and without PGP had a slower gait speed, with a small difference in speed between these two groups, which was however not significant. A study by Wu et al. (2008), showed that women with PGP tended to be afraid to move. Gait velocity was found to decrease with increasing kinesiophobia, with a correlation r = −0.64 between this two parameters (Wu et al. 2008). This slower gait has been linked to a quest for maximum stability and safety (Dingwell and Marin 2006; England and Granata 2007). In the light of Wu’s results, the present study expected the difference in speed between both groups of pregnant women to be much higher. However, in our case, women with PGP did not walk with smaller steps or decrease their gait speed, despite the pain. In addition, in women with PGP gait phases were altered when compared to non-pregnant women, which was characterized by increased stance and double support phases for women with PGP . A study by Bertuit et al. (2015) showed the same changes in gait phase between healthy pregnant women and non-pregnant women. The comparison with the control group highlights similarities between healthy pregnant women and those with PGP. Both groups of pregnant women displayed overall similar gait adaptations when compared to non-pregnant women but some differences were found between the two groups of pregnant women. Compared to healthy pregnant women, pregnant women with PGP displayed a decreased stance phase and increased single support phase. It is likely that these changes are the result of gait adjustments aimed at a reduction of shear stress in the pelvic joints and reactivation of the muscular work during weight transfer from one limb to the other (Vleeming et al. 2012; Krkeljas 2017). Balance, during pregnancy, does not seem to be affected by PGP because no differences were observed for step width. This parameter is strongly correlated with stability (Jang et al. 2008). This suggests that women with PGP, despite the pain and a potential instability of the pelvic girdle, do not display global instability or balance changes leading to specific adaptations of spatial and temporal gait parameters. These observations are to be related to the weak intra-individual variability measured for pregnant women with PGP, which does not differ either from healthy pregnant women or non-pregnant women. Wu et al. (2008) observed a greater variability of gait parameters in women with PGP, suggesting a wider individual variability in the adopted stabilization strategies. In this respect, a further hypothesis can be put forward: the low variability suggests a good reproducibility of gait for each woman. This regularity of gait can be a form of strategy or a gait adaptation for pregnant women who are careful to reproduce their specific gait pattern in order to avoid pain and to ensure the stability of gait.

We found no differences in temporal and spatial parameters between women with PGP who used a pelvic belt and those who did not. However, those who wore a belt displayed significant changes in a number of gait parameters between the two evaluations at T1 and T2. It can be hypothesized that these changes might not be related to pregnancy because they did not occur in group B. Due to the small size of this group, this hypothesis needs to be verified by future studies. The preferred gait velocity decreased by 8% between the first and second measurement, from 1.02 ± 0.23 m/sec to 0.93 ± 0.17 m/sec. Several hypotheses could be put forward. First, mass gain might influence this parameter. We know that mass gain is important in the third trimester of pregnancy. In our sample, the average mass gain was 12 ± 4 kg for group A. However, in our sample (groups A + B) only a negligible correlation was found (r = 0.27) between mass gain and gait velocity. Secondly, the pelvic belt could compress soft tissues in the pelvic girdle area and consequently stimulate proprioceptive receptors, which were identified in the SI ligaments (Vilensky et al. 2002; Varga et al. 2008). Force closure (ligaments and muscles) combined with pelvic extrinsic compression could stimulate these receptors known to regulate the neuromotor mechanisms controlling dynamic stability (Vilensky et al. 2002). Considering that the belt would add additional stability to posture, and pelvic girdle and stimulated the muscular work needed to keep pelvis level, these factors could play a significant role in changes in gait as a result of belt wearing (Krkeljas 2017). Moreover, the posterior-superior part of the SIJ is located at a depth of 5 to 7 cm under the skin. Therefore, the underlying tissues are probably deformed when a pelvic belt is applied. Depending on the direction and magnitude of the deformation, receptors in the skin, muscles, ligaments and the joint capsule may be stimulated (Shaffer and Harrison 2007). Proprioceptive receptors help to keep optimal postural control and perform accurate movements through perfect motor control (Shaffer and Harrison 2007). With more proprioceptive inputs, women wearing a belt may adopt natural and appropriate motion patterns for daily activities including walking. On the contrary, women not wearing a belt may use less appropriate stabilization or pain avoidance mechanisms, which could lead to abnormal stresses and eventually to pain. Indeed, pelvic pain starts mainly in the evening, following activities undertaken during the day (morning, afternoon) (Bertuit et al. 2017).

Pregnant women increased their step width by 1 cm to 2 cm. When a belt was worn during several weeks, step width increased again by 1 cm to 2 cm. In our study, certain pregnant women with PGP wearing a belt reached a step width of 12 cm at the end of pregnancy. The toe in/out angle followed a similar trend. It increased by 1 degree in pregnant women and it increased again by 1 degree in pregnant women with PGP. All these changes may again be attributed to the proprioceptive effect or to improved muscle activation (Krkeljas 2017). In pregnant women with PGP who wore a belt, single support and stance phase changed between the beginning and the end of the third trimester of pregnancy. Therefore, we can hypothesize that the use of a pelvic belt helped to stabilize the different gait cycle phases and to bring data closer to that of healthy pregnant women. It has been suggested that belts help promote the ‘self-locking’ mechanism and therefore pelvic stability, by a biomechanical effect increasing force closure. They facilitate mass transfer from one leg to the other during gait. The increase of the stance phase can be related to the reduction of shear forces facilitated by the belt (Vleeming et al. 2012).

No large difference between belt types appeared in this study. The gait parameters which changed between the seventh and ninth month of pregnancy changed for both belts and therefore did not appear to be influenced by the type of belt used. However certain small differences emerged. Belt 2 wearers displayed a decrease in gait velocity, single support time, and step length, while stance phase and step width increased. The width of this belt could explain the changes in these parameters. On the contrary, belt 1 being thinner was associated with less changes and accentuated less the effects of pregnancy on gait parameters. Therefore, belt 1 seems more appropriate for facilitating motor tasks.

For clinical practice, pelvic belts decrease pelvic girdle pain and improve functional capacity such as gait during pregnancy (Bertuit et al. 2017). A belt could influence musculoskeletal structures, by improving posture and pelvic stability, influencing gait parameters and facilitating motor and control function. Pelvic belts are easy to use and well accepted by women. This study encourages clinicians to suggest the use of pelvic belts to pregnant women suffering from PGP.

This study has several limitations: our group of healthy pregnant women was recruited during pre-natal gymnastics sessions. This suggests that these women were able to move freely and had a correct level of activity and knowledge of their body map. Therefore, our sample may not correctly represent the general population of pregnant women. This could induce a bias in our results by overestimating the abilities of this group. Although the number of drop-outs was acceptable over the entire sample of participants (8.6%), this rate was larger in the subgroup B – patients with PGP not wearing a belt during pregnancy. The main reason provided by the participants was a lack of motivation. Consequently, the limited size of group B influenced the effect sizes and the power of the study.

## Conclusion

Pregnant women with PGP showed nearly the same gait adaptations as healthy pregnant women, when compared to non-pregnant women. Pain induced a small modification of temporal and spatial gait parameters, but did not seem to affect global instability. Indeed, women with PGP, despite pain and potential pelvic girdle instability, did not display signs of global instability. The pelvic belts could have an effect on the gait of pregnant women with PGP through proprioceptive and biomechanical effects. A narrow and flexible belt could be more appropriate for facilitating the motor task, compared to a broad and rigid belt.
